# Characterization of a thermostable protease from *Bacillus subtilis* BSP strain

**DOI:** 10.1186/s12896-024-00870-5

**Published:** 2024-07-15

**Authors:** Tanveer Majeed, Charles C. Lee, William J. Orts, Romana Tabassum, Tawaf Ali Shah, Yousef A. Bin Jardan, Turki M. Dawoud, Mohammed Bourhia

**Affiliations:** 1 Department of Biotechnology, Kinnaird College for Women, Lahore, Punjab 54000 Pakistan; 2 National Institute for Biotechnology and Genetic Engineering (NIBGE), P.O. Box 577, Jhang Road, Faisalabad, Pakistan; 3 Bioproducts Research Unit, USDA-ARS, 800 Buchanan St., Albany, CA 94710 USA; 4 College of Agriculture Engineering and Food Sciences, Shandong University of Technology, Zibo, 255000 China; 5 Department of Pharmaceutics, College of Pharmacy, King Saud University, P.O. Box 11451, Riyadh, Saudi Arabia; 6 Department of Chemistry and Biochemistry, Faculty of Medicine and Pharmacy, Ibn Zohr University, Laayoune, 70000 Morocco; 7 Department of Botany and Microbiology, College of Science, King Saud University, P. O. BOX 2455, Riyadh, 11451 Saudi Arabia

**Keywords:** *Bacillus subtilis* BSP, Detergent/organic solvent-tolerant, Response surface methodology, Statistical optimization

## Abstract

This study used conservative one variable-at-a-time study and statistical surface response methods to increase the yields of an extracellular thermostable protease secreted by a newly identified thermophilic *Bacillus subtilis* BSP strain. Using conventional optimization techniques, physical parameters in submerged fermentation were adjusted at the shake flask level to reach 184 U/mL. These physicochemical parameters were further optimized by statistical surface response methodology using Box Behnken design, and the protease yield increased to 295 U/mL. The protease was purified and characterized biochemically. Both Ca^2+^ and Fe^2+^ increased the activity of the 36 kDa protease enzyme. Based on its strong inhibition by ethylenediaminetetracetate (EDTA), the enzyme was confirmed to be a metalloprotease. The protease was also resistant to various organic solvents (benzene, ethanol, methanol), surfactants (Triton X-100), sodium dodecyl sulfate (SDS), Tween 20, Tween-80 and oxidants hydrogen per oxide (H_2_O_2_). Characteristics, such as tolerance to high SDS and H_2_O_2_ concentrations, indicate that this protease has potential applications in the pharmaceutical and detergent industries.

**Supplementary Information:**

The online version contains supplementary material available at 10.1186/s12896-024-00870-5.

## Introduction

Proteases are a distinct group of enzymes that are frequently used in a series of industries, containing food, detergent, clothing, and pharmaceutical. Because of their rapid growth and high capacity for protein production, microorganisms such as *Bacillus* sp. are the main sources of proteases [1]. Due to the increasing demand for proteases for varied applications, there is a continuous need to discover new versions of these industrially important enzymes.

Thermophilic microorganisms are known to be excellent sources of thermostable proteases [2]. Enzymes from thermophilic microorganisms exhibit faster reaction rates at high operating temperatures and are noticeably more stable than similar enzymes from mesophilic species. Several thermophilic bacterial strains, such as *Bacillus licheniformis*, *B. stearothermophilus*, and *B. pumilus*, have been explored for protease production [3]. There has been much research and development focused on the fermentation process to optimize high protease yields. Engineers in the biochemical and process industries can create scale-up plans for better enzyme production by utilizing statistically designed tests [4].

Protease commercial manufacturing is still in its early stages in developing countries like Pakistan for its use in various productions, including the leather and detergent sectors. In this study, a new thermophilic bacterial strain was isolated and screened for proteases. The strain, *B. subtilis* BSP, was found to express a thermostable protease, and was characterized. Using classical one-variable time study and statistical response surface methodology (RSM) methodologies, the physical and nutritional parameters were changed to maximize the production of the protease enzyme. Additionally, the protease was biochemically characterized to determine metal cofactor requirements and resistance to detergents and organic solvents. This strain, *B. subtilis* BSP express protease enzymes, which highly stable and resistant to various organic solvents, and surfactants and hold great potential for new uses in many industries.

## Materials and methods

All chemicals were of analytical-reagent grade or higher quality and were purchased from Sigma-Aldrich (St. Louis, MO, USA) unless otherwise stated. Caseins were obtained from Lab M Limited (UK) and Sigma Aldrich (USA). Tryptone, agar-agar, yeast extract (YE), and peptone were purchased from Acumedia (Michigan, USA). Enzymes were obtained from Fermentas (St. Leon-Rot, Germany) and New England Biolabs (Ipswich, MA, USA). A Perkin Elmer GeneAmp PCR System 2400 Thermal Cycler (CT, USA) and thin-wall PCR tubes (Multi-ultra tubes, 0.2 mL, Carl Roth, Karlsruhe, Germany) were used for all PCRs.

### Screening, growth conditions, and selection

Thermophilic bacterial strains were isolated from soil following enrichment in the basal salt medium 1 g/L (NH_4_)_2_PO_4_, 1 g/L K_2_HPO_4_, 0.5 g/L MgCl_2_, 10 g/L casein, and 10 g/L yeast extract, followed by plating on the same medium supplemented with 1.5% agar at 60^◦^C. The bacterial cells were stored at -80 °C in a (50% v/v) glycerol solution, while the bacterial cell culture was maintained on Luria Broth agar plates wrapped in Parafilm at 4 °C. Culture cells that made clear halos in the casein substrate were isolated and selected. Genomic DNA was extracted from bacterial isolates using the CTAB (Cetyl Tri-methyl Ammonium Bromide) method. The bacterial cells were cultured in LB medium overnight at 37 °C and subsequently harvested by centrifugation at 7500 x g. The cells were then re-suspended in 5 mL of T.E buffer, to which 20 mg of lysozyme was added. The suspended cells were incubated at 37 °C for 5 min. Following this, 500 µL of 10% SDS, 25 µL of proteinase K (25 mg/mL), and 3 µL of RNase were added to the incubated suspension. The contents were thoroughly mixed and incubated at 37 °C for 10 min. After incubation, 0.9 mL of 5 M NaCl and 0.75 mL of NaCl/CTAB were added and mixed thoroughly. The mixture was then incubated at 65 °C for 20 min. The protein contents were extracted twice with an equal volume of Phenol-chloroform-isoamyl alcohol (25:24:1) and subsequently centrifuged for 10 min at 7500 x g and 4 °C to separate the organic and aqueous phases, with the DNA contained in the aqueous phase. The aqueous supernatant was transferred to a fresh tube, and 0.6 volume of isopropanol was added and mixed until a white DNA thread precipitated out of solution and condensed into a tight mass. The DNA was pelleted by centrifugation at 7500 x g for 5 min, and the supernatant was discarded. Finally, the pellet was washed twice with 70% ethanol and dried at room temperature. The dried pellet was then re-suspended in 100 µL of T.E for further use. The total genomic DNA was amplified for 16 S rRNA genes with the following universal primers-.

$$FD1(5^{\prime}AGAGTTTGATCCTGGCTCAG3^{\prime})$$

$$RP1(5^{\prime}ACGG(H)TACCTTGTTACGACTT3^{\prime})$$

The genomic DNA was amplified in the polymerase chain reaction (PCR) under the following conditions: an initial denaturation step at 95 °C for 5 min, followed by 30 cycles of denaturation at 95 °C for 30 s, annealing at 53 °C for 30 s, and extension at 72 °C for 5 min. Subsequently, a 2 µL aliquot of the reaction mixture was loaded onto a 1% agarose gel for electrophoresis to confirm the presence of the PCR band. The amplified 16 S DNA sequence was then determined using automated DNA sequencing. The resulting sequence was subjected to analysis using the BLAST resource website provided by the National Center for Biotechnology Information (NCBI) (https://blast.ncbi.nlm.nih.gov/Blast.cgi), and homologous sequences from related species were utilized for the construction of neighbor-joining trees.

### Protease production and assay

For the production of protease, a colony of *Bacillus subtilis* BSP was inoculated a 250 mL flask containing sterile 100 mL of basal medium [pH 7.0 ± 0.2] [1.0 g/L K2HPO4, 1.0 g/L MgSO4.7H2O, 5.0 g/L NaCl, 1.0 g/L Peptone, 1.0 g/L Yeast extract]. The medium was incubated at 37 °C in a shaking incubator at 150 rpm. The fermented broth was centrifuged at 10,000 rpm at 4 °C for 10 min. The supernatant was used for protease activity. The Lowry method, with minor changes, was used to carry out protease activity assays [5]. The protease received a 30-minute preincubation at 50 °C. One mL of 1% casein solution [1% casein dissolved in Tris-HCl buffer, 50 mM, pH 9.0] was added to the 1 mL crude protease to start the reactions, which were then incubated for 20 min at 50 °C. A 3 mL of 5% trichloroacetic acid (TCA) was added to stop reaction and the sample mixture was centrifuged. Following a 30-minute incubation at room temperature, the precipitate was removed by centrifugation, and the optical density of the assays was subsequently measured at a wavelength of 660 nm (UV-1800 Spectrophotometer, Shimadzu, Japan). A series of solutions ranging from 0 to 60 µg/mL tyrosine were prepared to make a standard curve. The enzyme unit was calculated for the assay condition as such that, one unit of protease activity was determined as the amount of protease required to release 1 µg/mL tyrosine from casein in 1 min under assay conditions.

### Screening of significant culture parameters by conventional methodology

Initially, a classical one variable at a time (OVAT) strategy was used to adjust factors impacting bacterial growth and enzyme production in a submerged fermentation system by maintaining all other components in the growth medium at constant levels. In triplicate, 250 mL Erlenmeyer flasks with 50 mL of bacterial culture suspension were used for each experiment. The following variables were examined: temperature range between 40 and 90 °C, 0.5-3% casein content, medium pH (3.0–10.0) [pH 0.1 M sodium acetate for pH 4.0–5.0, 0.1 M potassium phosphate for pH 6.0–9.0), and 0.1 M glycine-NaOH buffer for pH 10.0–11.0)], and fermentation time (12–72 h). The average values of all experiments were reported.

### Optimization by statistical response surface methodology (RSM)

Later, a statistical Box Behnken design using statistical software (Design-Expert 9.0.3.1; Stat Ease, Inc., MN, USA) was employed to further improve protease production [6]. The focus of this analysis was on the optimization of cultivation conditions to maximize *Bacillus* sp. BSP protease production is based on the interactions of four variables: fermentation time (A), pH (B), casein concentration (C), and inoculum volume (D). With 29 experimental setups (Table 1), the impact of these variables on enzyme production was examined at three levels (-1, 0, and + 1 as minimal, center, and maximal values). The experiments were conducted in a 50 mL basal salt medium contained in 250 mL Erlenmeyer flasks. After the *Bacillus* sp. BSP was inoculated into the growth media, the cultures were shaken (180 RPM) at 60 °C. The level of protease produced was quantified after the fermentation period using the protease assay described above. The average data from each experiment were reported, and each experiment was done in triplicate. The expected response was calculated using the second-order polynomial Eq. (1), and the data were then fitted to the multiple regression equation, which revealed the following empirical model equation:

1 $$Y = {\beta _0} + \sum \;{\beta _n}{X_n} + \sum \;{\beta _{nn}}{X_n}^2 + \sum \;{\beta _{nm}}{X_n}{X_m}$$

Where, Y represents the predicted response

β_0_ is the offset term

β_n_ is the linear coefficient

β_nn_ is the squared coefficient

β_nm_ is the interaction coefficient

X_n_ and X_m_ are the independent variables

X_n_^2^ is the squared effect

X_n_X_m_ is the interaction effects

The resulting model equation for this four-variable system was:

2 $$\begin{array}{l}Y = {\beta _0} + \;{\beta _1}A + \;{\beta _2}B + {\beta _3}C + {\beta _4}D + {\beta _{11}}{A^2} + \\{\beta _{22}}{B^2} + \;{\beta _{33}}{C^2} + \;{\beta _{44}}{D^2} + \;{\beta _{12}}AB + \;{\beta _{13}}AC + \;\\{\beta _{14}}AD + \;{\beta _{23}}BC + \;{\beta _{24}}BD + \;{\beta _{34}}CD\end{array}$$

### Purification of proteases

1 L of culture was cultivated for 72 h at 60 °C and pH 8 to produce protease enzyme. Centrifugation was used to separate the culture supernatant from the growth medium, and then ammonium sulfate precipitation (at 70% saturation) was used. The precipitate was obtained by centrifuging it at 7500 rpm for 20 min at 4 °C, and the pellet that was produced was then resuspended in 10 mL of ice-cold, pH 7.5, 0.1 M phosphate buffer. By dialyzing against a 0.1 M phosphate buffer with a pH of 7.5, the ammonium sulfate was eliminated. A DEAE-cellulose column from Sigma-Aldrich in Missouri, USA, was used to bind the dialyzed product, and a solution of 0.5 M NaCl and 0.1 M phosphate buffer, pH 7.5, was used to elute the protease at a flow rate of 0.8 mL/min. Using the Bradford assay technique, the protein concentration was determined [7]. The samples having the highest protease activity were combined and applied at a flow rate of 0.5 mL/min to a Sephadex G-200 column (Sigma-Aldrich) that had been pre-equilibrated with 0.1 M phosphate buffer, pH 7.5.

### Determination of pH and temperature effects

Protease activity was tested against 1% casein substrate dissolved in a variety of buffers (0.05 M) over a wide pH range (3.0–11.0), including 0.1 M sodium acetate (pH 4.0–5.0), 0.1 M potassium phosphate (pH 6.0–9.0), and 0.1 M glycine-NaOH buffer (pH 10.0–11.0). The substrate and reaction mixtures were combined, and the temperature was maintained at 50 °C for 20 min. By 20 min of assaying against a pre-heated 1% casein substrate at various temperatures (40, 50, 60, 70, 80, and 90 °C), the ideal temperature for maximal protease activity was determined. Protease was subjected to a range of temperatures (40–90 C) for 1 h in order to test its thermostability. Next, after 20 min at 50 degrees Celsius, the heat-treated enzyme was tested against 1% casein substrate. The Lowry method, which has been previously described, was used to calculate the enzyme activity levels from the pH optimization, temperature optimization, and thermostability studies.

### Determination of organic solvents, surfactants, and oxidant effects

Several organic solvents (DMSO, methanol, ethanol, benzene, and hexane), H_2_O_2,_ and surfactants (sodium dodecyl sulfate (SDS), Triton X-100, Tween 20 and Tween-80 were examined for their effects on protease activity. A 10% (v/v) final concentration of these substances was added to 1 mL of a protease enzyme solution, and the mixtures were then incubated at 50 °C and pH 8.0. Surfactants and H_2_O_2_ were incubated for 30 min whereas organic solvents were incubated for 10 min. One mL of 1% casein substrate was added after the incubation period, and the protease assay was carried out for 20 min at 50 °C. The Lowry approach, which is previously described, was used to determine the enzyme activity of the protease.

### Determination of metal ions and inhibitors effects

The impacts on protease activity by metal ions (Co^2+^, Fe^2+^, Ca^2+^, Mn^2+^, Cu^2+^, Ni^2+^, Mg^2+^, and Zn^+ 2^) and inhibitors iodoacetamide (IAA), β-mercaptoethanol (β-ME), dithiothreitol (DTT), and ethylenediaminetetraacetate (EDTA), phenylmethylsulfonyl fluoride (PMSF) were studied. At 50 °C, the metal ions and protease enzyme were incubated for 30 min. At 37 °C for 30 min, the protease enzyme and inhibitors were incubated. All of the cations and inhibitors were used in a 1 mL volume having a 5 mM final concentration. The enzyme activity was started after 1 mL of 1% casein substrate addition, and incubated for 20 min at 50 °C, the proteolytic activity was calculated as previously described [8]. Protease that hadn’t been altered served as the control reaction, which was standardized to 100% activity. The tested cations corresponded to the following salts: CaCl_2_, CuCl_2_, CoCl_2_, MnSO_4,_ MgCl_2_, FeSO_4_, NiCl_2_, and ZnCl_2_.

## Results and discussion

### Screening for proteolytic activity

Thermophilic bacterial strains demonstrating protease activity were isolated from municipal soil waste. Pure strains that produced protease activity were selected by screening for colonies that generated large clearings on agar plates containing 1% casein at 60^◦^C. These strains were analyzed further for the capacity to secrete protease activity into liquid culture containing 1% casein at 60^◦^C. The thermophilic BSP isolate was found to demonstrate some of the highest protease activity. This strain was characterized as an aerobic, motile, rod-shaped, Gram-positive, catalase-positive, indole-negative bacterium. Based on these characterizations, this isolate was designated as *Bacillus subtilis* BSP, a thermophilic protease producer. This strain was picked for additional research on protease characterization, scaling up, and production.

**Fig. 1:**
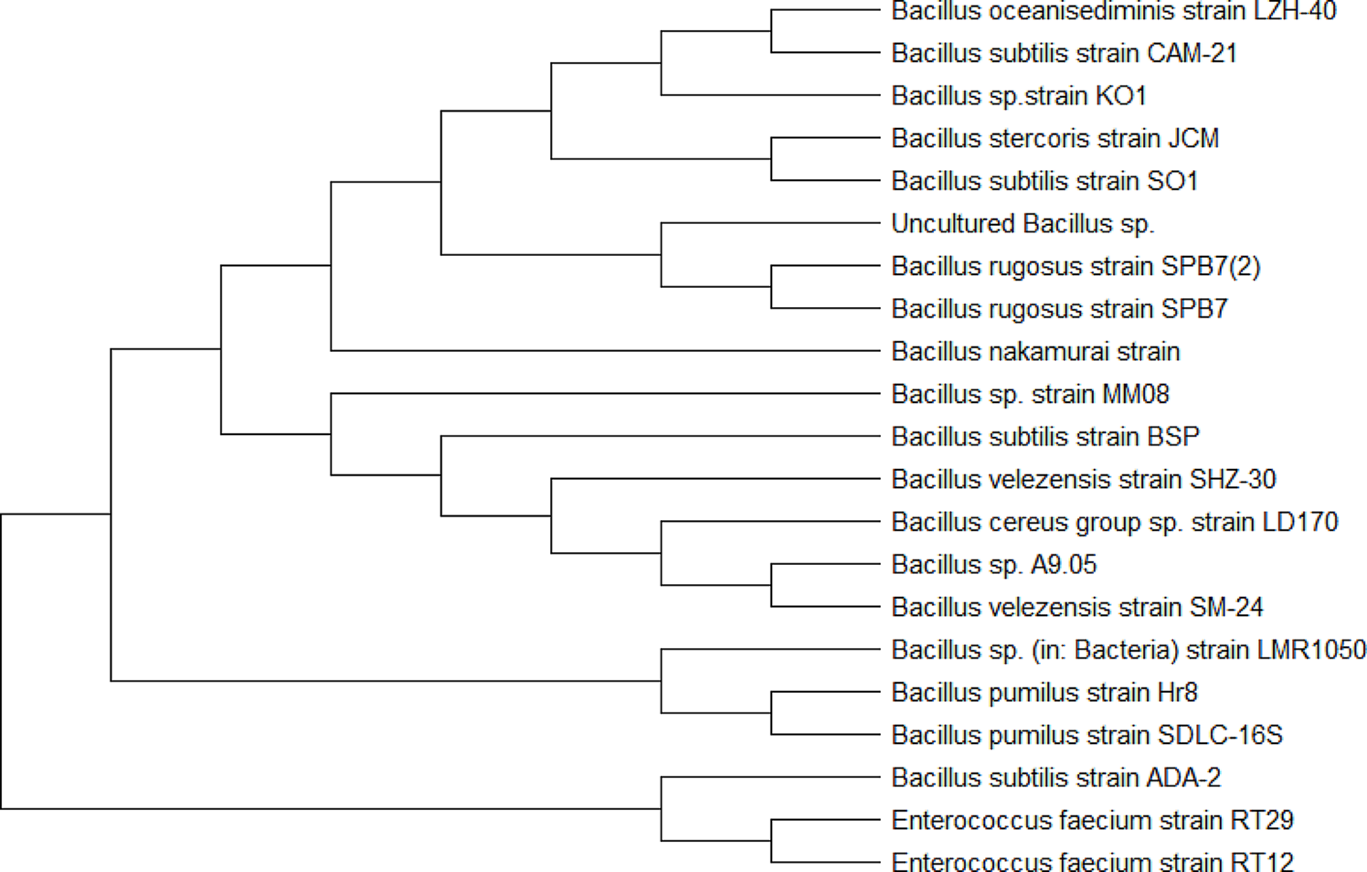
Neighbor joining tree showing the phylogenetic relationship of the *Bacillus subtilis* BSP strain with the related species based on the 16sRNA sequences

The 16 S rRNA gene sequence was sequenced and submitted to GenBank (accession number EF644419.1). The phylogenetic tree is shown in Fig. 1. The 16sRNA gene sequence similarity demonstrated that this strain belongs to various *Bacillus sp.* strains with 98–99% similiarity. The close matching strains were *Bacillus subtilis* strain ADA-2, *Bacillus nakamurai* strain, *Bacillus oceanisediminis* strain, *Bacillus velezensis* strain SHZ-30, *Bacillus cereus* strain LD170, *Bacillus pumilus* strain Hr8, *Bacillus velezensis* strain SM-24, *Bacillus stercoris* strain JCM and *Bacillus rugosus* strain SPB7.

### Screening of significant factors using a conventional approach

**Fig. 2:**
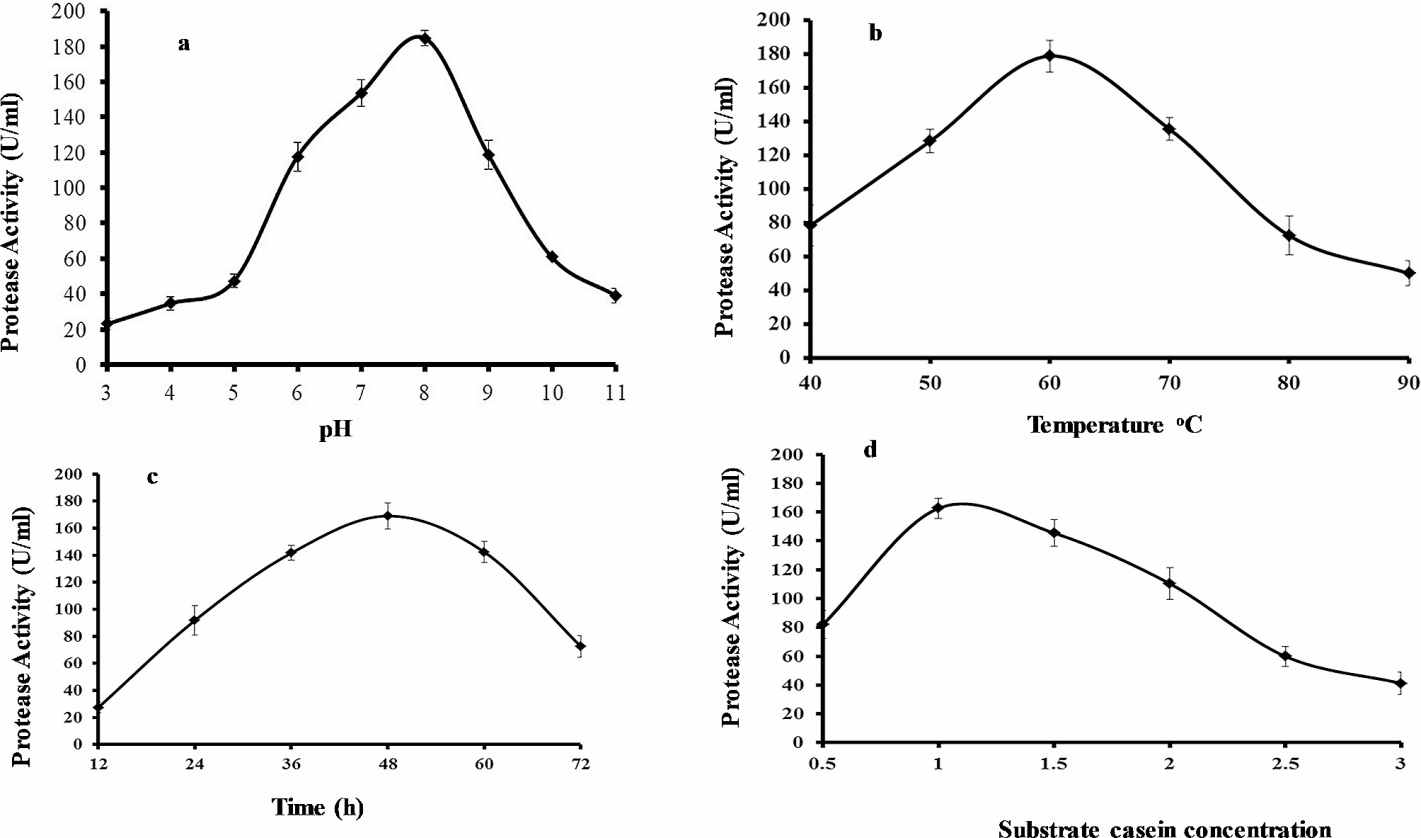
Results of experimental conditions on protease production by *Bacillus subtilis* BSP. (**a**) Effect of initial pH after 48 h fermentation at 60^o^C. (**b**) Effect of temperature after 48 h fermentation at pH 8.0. (**c**) Effect of fermentation time at pH 8.0 and 60^o^C. (**d**) Effect of casein concentration after 48 h fermentation at pH 8.0 and 60^o^C

The one variable at a time (OVAT) methodology was run to check the effects of initial pH, fermentation time, temperature, and casein concentration upon protease production in shake flasks. Protease activities may differ depending on pH levels. Each protease has a specific pH range for optimal activity. It is noteworthy that pH sensitivity of protease activity can differ depending on protease type and substrate used. Therefore, determining the optimal pH range for a specific protease is crucial to obtain maximum activity. In this study, the protease was highly active between pH 3 and 11. The optimal pH for protease production was determined by using 1% casein to induce enzyme production and varying the initial pH (3.0–11.0) of the growth medium containing 1% casein at 60^◦^C (Fig. 2a). *B. subtilis* BSP was capable to synthesize and express protease across a different choice of culture pH (3.0–9.0), whereas the pH 8.0 resulted in the highest protease production (184 U/mL). At pH 10.0, the enzyme level was dramatically decreased. *B. subtilis* BSP was also found to produce protease enzyme across all the temperatures studied (40˚C to 90˚C) at pH 8 and 1% casein as substrate (Fig. 2b); however, 60˚C was the optimum temperature for protease production (179 U/mL). Previously it is reported that the protease is active at pH levels ranging from 5 to 11. Similarly, another specific protease has been found to be active between pH 2 and 12. The optimum pH and temperature values reported in this study for protease production are similar to those of other thermophilic *Bacillus* sp [9, 10].

Tracking protease production across various time points indicated that a fermentation time of 48 h led to the highest enzyme production (179 U/mL) (Fig. 2c). Protease levels decreased from 60 to 72 h. Previous reports of *Bacillus* sp. GUS1 and *Bacillus* sp. P003 showed that these strains had maximum protease production during the stationary phase of growth [11]. In addition, optimum production occurred at 36 h for *Bacillus* sp. subtilisin protease and 72 h for *Bacillus licheniformis* (MTC NO. 7053) alkaline protease [12–14].

The composition of the growth medium was optimized by varying the concentration of the casein substrate which serves as a protease production inducer. Optimum enzyme production (165 U/mL) was observed when using 1% casein (Fig. 2d). A lower casein concentration (0.5%) reduced protease production while the maximum substrate concentration tested (3%) resulted in the lowest protease production.

### Statistical Box-Behnken design matrix, model fitting, and analysis of variance (ANOVA)

To further optimize protease production, a response surface methodology (RSM) was employed. A Box-Behnken design with four factors was utilized to fit a second-order polynomial model, which resulted in 29 experimental setups to conduct for the optimization study. Table 1 demonstrates the experimental strategy and associated protease yields.

**Table 1:** Box Behnken experimental designs with the experimental and predicted values of protease production

Run Order	Factor A (Fermentation Time h)	Factor B (pH)	Factor C (Casein Conc. %)	Factor D (Inoculum Density %)	Observed Response	Predicted Response
1	48	7	2	1.5	163	166
2	72	8	3	1	229	228
3	48	8	3	1.5	232	235
4	72	8	2	1.5	260	256
5	24	8	2	0.5	228	231
6	24	8	3	1	225	225
7	24	7	2	1	164	162
8	72	8	1	1	262	263
9	24	8	2	1.5	195	194
10	48	9	1	1	209	211
11	24	8	1	1	209	211
12	48	8	2	1	279	281
13	48	8	2	1	278	281
14	72	9	2	1	199	201
15	72	8	2	0.5	221	222
16	24	9	2	1	177	176
17	72	7	2	1	189	190
18	48	7	1	1	169	169
19	48	9	3	1	173	171
20	48	8	1	1.5	223	221
21	48	8	1	0.5	250	247
22	48	9	2	0.5	181	179
23	48	8	2	1	295	281
24	48	8	3	0.5	210	212
25	48	7	3	1	191	188
26	48	9	2	1.5	191	191
27	48	7	2	0.5	180	180
28	48	8	2	1	280	281
29	48	8	2	1	272	280

Box–Behnken design was used to evaluate the collective effects of 04 parameters (pH, fermentation time, inoculum density, and substrate concentration) on *B. subtilis* BSP protease secretion. Table 2 represents the design with three replicates showing the predicted responses based on a polynomial equation. ANOVA is a statistical technique used to determine the significance of a proposed model and its parameters. In general, higher F-values and lower p-values are indicative of significant coefficient terms [15]. ANOVA analysis suggested a regression equation with the terms of protease activity (Y) as a response to inoculum density (D), substrate concentration (C), pH (B), and fermentation time (A) as follows:

3 $$\begin{array}{l}Y = 280.88 + 13.46A + 6.22B - 5.19C - 0.57D - \\0.72AB - 2.29AC + 17.80AD - 14.56BC + 6.50BD + \\12.30CD - 26.08{A^2} - 72.71{B^2} - 23.17{C^2} - 29.00{D^2}\end{array}$$

**Table 2:** ANOVA for response surface quadratic model

Source	Sum of squares	df	Mean square	F-Value	Prob > F
Model	43212.23	14	3086.59	114.27	< 0.0001
D-Inoculum density	3.94	1	3.94	0.15	0.7082
C-Substrate	323.03	1	323.03	11.96	0.0038
B-pH	464.07	1	464.07	17.18	0.0010
A-Time	2173.22	1	2173.22	80.46	< 0.0001
AB	2.07	1	2.07	0.077	0.7861
AD	1266.90	1	1266.90	46.90	< 0.0001
AC	604.62	1	604.62	22.38	0.0003
CD	604.84	1	604.84	22.39	0.0003
BC	848.29	1	848.29	31.41	< 0.0001
BD	169.00	1	169.00	6.26	0.0254
D^2^	5456.22	1	5456.22	202.01	< 0.0001
C^2^	3483.35	1	3483.35	128.96	< 0.0001
B^2^	34288.62	1	34288.62	1269.47	< 0.0001
A^2^	4411.81	1	4411.81	163.34	< 0.0001
Lack of Fit	88.58	10	8.86	0.12	0.9409
Residual	378.14	14	27.01		
Cor Total	43590.37	28			
Pure Error	289.56	4	72.39		
Coefficient of variation (C.V. %)	2.38	Pred R-Squared	0.9779		
Mean		218.41	Adj R- Squared		0.9827
Standard deviation		5.20	R-Squared		0.9913
PRESS		962.67	Adeq Precision		31.886

A linear model’s percentage of variation in the response variable is shown by the R-Squared (coefficient of determination). R-Squared is a measure of how well a model matches experimental data [16]. Model fit is thought to be good when the R-Squared value is at least 0.8. R-Squared values of 0.9913 and 0.9827 for the adj R-Squared value in this analysis showed that the proposed response model can describe the reaction very well at a 95% confidence level. A difference between Pred R-Squared and Adj R-Squared of less than 0.2 is ideal, and this desirable state is reflected in the values of Pred R-Squared (0.9779) and Adj R-Squared (0.9870). The Adeq Precision ratio (signal-to-noise ratio), which in this case was 31.886, illustrates the model’s good fit. A ratio above 4 is ideal. With a noise-related error chance of only 0.01%, the F-value of 114.27 validates the model’s significance. The model terms are likely to be extremely important if the values for “Prob > F” is less than 0.0500. The linear fermentation time (A-Time), the interaction of the fermentation time and inoculum size (AD), the interaction of the pH and substrate concentration (BC), the fermentation time (A2), the pH (B2), the substrate concentration (C2), and the inoculum size (D2) were found to be significant model terms in this study (P0.0001) with a cutoff of *P* > 0.1000.

### Response surface analysis

**Fig. 3:**
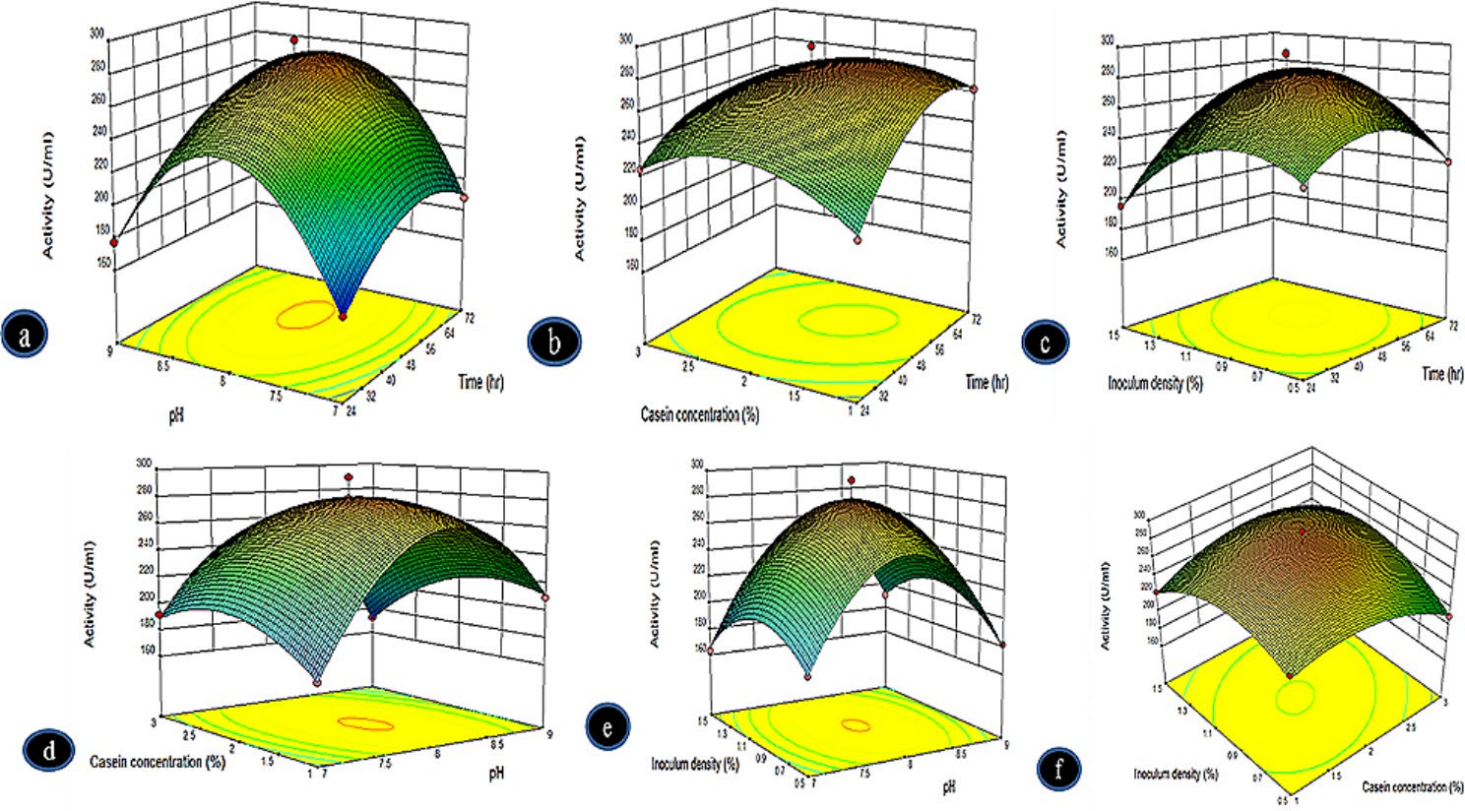
Response-surface plots of protease production *Bacillus subtilis* BSP showing mutual interactions

Three dimensional plots were obtained in order to study the effect of the combined relations of the factors being studied and to identify the optimal value of each variable at which maximum protease yield was achieved (Fig. 3). Figure 3a shows the effect of fermentation period and pH on protease production when inoculum density and casein concentration were kept constant. Protease yield increased with increasing pH up to pH 8.0 at the ideal fermentation period of 64 h. Protease production declined when the pH was more acidic or basic than pH 8.0 and when the fermentation time exceeded 64 h. The 3D response surface plot for the interaction of casein content and fermentation time is shown in Fig. 3b. The highest alkaline protease yield was achieved at a casein concentration of 2.5% and a fermentation time of 72 h. As displayed in Fig. 3c, the highest yield of alkaline protease occurred when the volume of inoculum density ranged from 0.9 to 1%, and the fermentation period was between 48 and 56 h. According to Fig. 3d, protease synthesis rose when the substrate concentration was increased to 2% and the pH was raised to 8.0. The impact of the combined interaction of pH and inoculum density on enzyme synthesis can be observed in Fig. 3e. Up to 1% inoculum densities and pH 8 resulted in higher protease yields. At inoculum densities exceeding 1% and pH levels above 8.0, the synthesis of enzymes dropped. The interaction effect of casein concentration (1–3%) and inoculum density (1–3%) on protease synthesis is shown in Fig. 3f. A 1.8% casein concentration and 1% inoculum density resulted in the highest enzyme yield. The quadratic mode equation was solved to get the best values of each variable for maximum protease activity. It was discovered that pH 8.0, 1% inoculum density, 72 h of fermentation, and 1% casein content were the best values for each variable. At these conditions, a response in protease activity of 295 U/mL was observed.

### Expression and purification of protease

Using the optimized parameters calculated from the RSM results, four 500 mL Erlenmeyer flasks each containing 250 mL media were used to produce protease enzyme.

At the harvesting point, fermented broth was centrifuged at 10,000 rpm for 10 min at 4^o^C and the enzyme activity was measured. The protease activity was increased 2.1 fold in the synthetic medium when compared to that of complex media. Among the complex media, the foxtail millet medium showed higher protease activity. Protease was partially purified from cell free filtrate of *Bacillus amyloliquefaciens* TSA-2 cultured by employing ammonium sulphate precipitation followed by dialysis. Maximum amount of protein was precipitated at 70–80% saturation of ammonium sulphate. The outcome of ammonium sulphate precipitation was 1.56- fold increase in purity and 80.5% yield of protease. When the precipitated protein was dialysed, the purification was further increased to 1.35 fold with 71.3% yield.

**Fig. 4:**
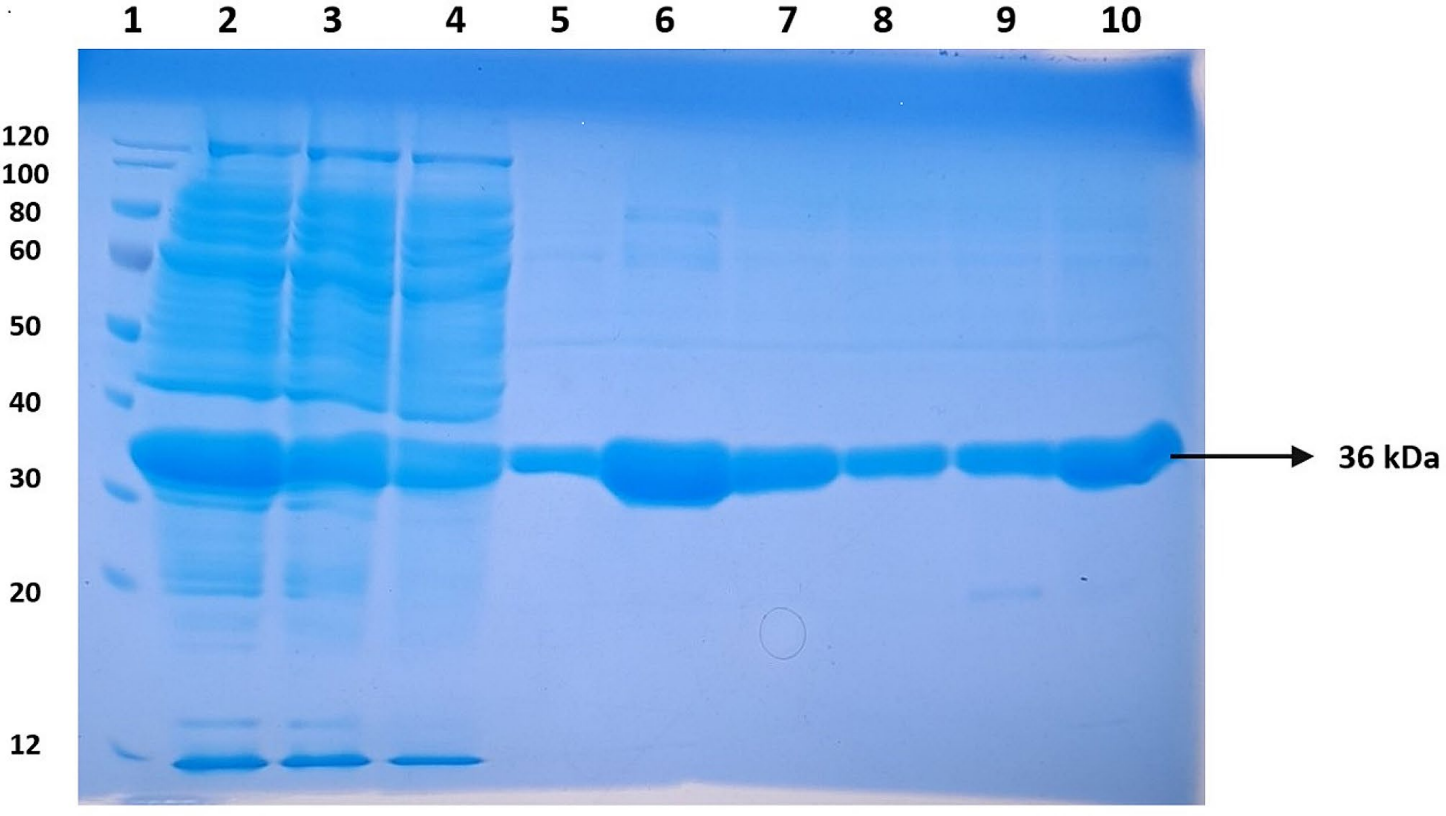
SDS–PAGE of the thermostable protease from *Bacillus subtilis* BSP. Lane 1, molecular weight markers; lane 2, culture supernatant; lane 3, dialyzed protease after ammonium sulfate precipitation; lane 4, protease purified from DEAE-cellulose column; and lane 5–10, Sephadex G-200 purified protease. 12–120 kDa Protein Marker, Catalogue No: abx098114

A protease enzyme exhibiting a specific activity of 39.3 U/mg was detected in the supernatant of a bacterial culture, which contained a crude enzyme with a protein concentration of 560 mg. This enzyme was subsequently enriched through ammonium sulfate precipitation. The resulting precipitate was then resuspended and the protein was further purified using DEAE-cellulose ion exchange chromatography. Following ammonium sulfate precipitation, the enzyme exhibited a specific activity of 58.2 U/mg with a protein concentration of 208 mg. Dialysis further increased the enzyme’s specific activity to 71.1 U/mg, with a concentration of 107 mg. The DEAE-cellulose ion exchange chromatography step yielded 36 mg of protein and resulted in an enzyme specific activity of 111.3 U/mg. After the partial purification fractions obtained from the Sephadex G-200 gel filtration column, the protein yield was 3.34 mg, and the enzyme’s specific activity was further enhanced to 180.2 U/mg. On SDS-PAGE, the isolated protease was present as a partially purified fractions, with a symmetrical 36 kDa band (Fig. 4). Many *Bacillus* species have been reported with variety of molecular masses for proteases: thermostable protease (49 kDa) from *Bacillus* strain HUTBS71 [17]; thermostable proteases of (36 kDa) from *Bacillus stearothermophilus* TLS33 [18]; *Bacillus licheniformis* VSG1has (40 kDa) [19]; (28 kDa from *Bacillus pumilus* MK6-5 [20]; metalloserine protease of (36 kDa) from *Brevibacillus thermoruber LII* [21]; and an alkaline serine proteases of (37–40 kDa) from thermophilic *Bacillus* sp. GUS1 [15, 22].

### Determination of optimum pH and temperature

**Fig. 5:**
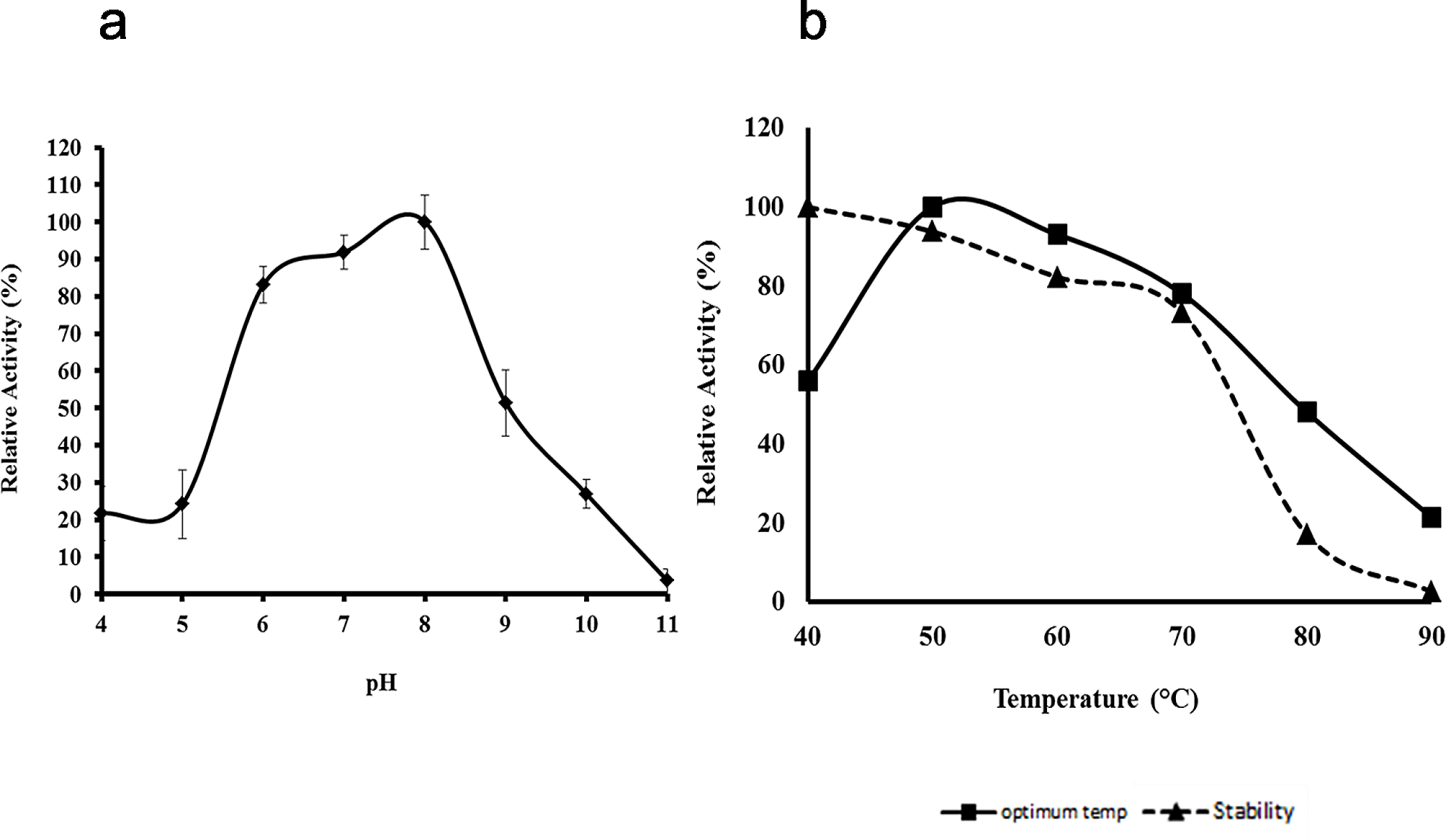
Effects of pH and temperature on protease activity. (**a**) Protease activity assay was conducted at various pH at 50 °C. (**b**) Protease activity assay was conducted at various temperatures at pH 8.0 (■). Protease was pre-incubated at different temperatures before assaying for residual activity at 50 °C at pH 8.0 (▲)

The enzyme activity was analyzed at various pH ranging from pH 3.0 to pH 11.0 (Fig. 5a). The maximum protease activity was observed at pH 8.0. The enzyme also had significant activity from pH 6.0 to pH 9.0 with 83% and 51% relative proteolytic activity at these conditions, respectively.

The protease activity was also investigated at various temperatures ranging from 40–90ºC at pH 8.0 (Fig. 5b). The optimum temperature for protease activity was 50ºC. The enzyme activity decreased at temperatures above 60ºC. The thermostability of the protease was examined by heat treating the protease at different temperatures (40–90ºC) without substrate for 60 min, and the relative residual activity was assayed at 50ºC (Fig. 5b). The protease had high stability at 40ºC and 50 °C and retained 73% of its original activity even after heat treatment at 70ºC. However, 84% of the enzyme activity was lost after incubation at 80ºC [23].

### Effect of metal ions, organic solvents, surfactants, and inhibitors on protease activity

Different metal ions (Co^+ 2^, Ca^+ 2^, Cu^2+^, Mg^+ 2^, Fe^+ 2^, Mn^+ 2^, Ni^2+^ and Zn^+ 2^), organic solvents (methanol, ethanol, benzene hexane, and dimethyl sulfoxide (DMSO)), various surfactants (Triton X-100, sodium dodecyl sulfate (SDS), Tween-20, Tween-80, H_2_O_2_, and potential inhibitors dithiothreitol (DTT), (β-mercaptoethanol (β-ME), iodoacetamide (IAA), ethylenediaminetetraacetate (EDTA), phenylmethylsulfonyl fluoride (PMSF)) were checked to analyze any effects that these reagents might have upon the *B. subtilis* BSP protease activity.

The enzyme activity was stimulated by the Ca^+ 2^ and Fe^+ 2^ cations (Table 3). In contrast, Cu^2+^ dramatically reduced protease activity to 23%. The protease activity was decreased to a lower extent by the Ni^+ 2^, Mg^+ 2^, Zn^+ 2^, and Co^+ 2^ cations. The Mn^+ 2^ cation was found to have little effect upon the enzyme activity. Similarly, a purified detergent-stable, solvent-tolerant protease STAP and a *Bacillus stearothermophilus* alkaline protease [24] were reported to also be stimulated by Ca^2+^ cation.

The *B. subtilis* BSP protease was found to be quite stable in the presence of many organic solvents (Table 3). The enzyme was tolerant in 10% benzene, ethanol, and methanol. Hexane had the greatest impact by reducing the enzyme activity to 76%. In addition, 10% DMSO reduced the enzyme activity to a lesser extent (83%).

The *B. subtilis* BSP protease also retained activity in the presence of many surfactants (Table 3). The protease maintained 97% of its activity in 10% (v/v) Triton X-100. A similar degree of stability against this detergent was reported for the *Bacillus pumilis* KS12 keratinase [25]. The *B. subtilis* BSP protease activity was decreased to a slightly greater degree by 10% H_2_O_2_. Interestingly, the protease activity was enhanced to 112% by the presence of 10% SDS. This is the first report of an SDS-stimulated protease from a *Bacillus* sp. The fact that the enzyme was tolerant to the tested surfactants and oxidants indicates that the structure of the protein is likely well-packed with rigid native conformation.

**Table 3:** Effect of metal ions, organic solvents, surfactants and H_2_O_2_ on protease activity

Metal Ion	Concentration (mM)	Relative Activity (%)
Control	-	100
Ca	5	122.4
Co	5	76.5
Cu	5	22.7
Fe	5	113.5
Mg	5	66.7
Mn	5	104.5
Ni	5	55.5
Zn	5	63.5
**Surfactant**	**Concentration (%)**	**Relative Activity (%)**
Control	-	100
SDS	10	112
Triton X-100	10	97.3
Tween-20	10	105.2
Tween-80	10	87.6
H_2_O_2_	10	95.4
**Organic Solvent**	**Concentration (%)**	**Relative Activity (%)**
Control	-	100
Ethanol	10	95.77
Benzene	10	95.8
DMSO	10	83.2
Hexane	10	75.6
Methanol	10	92.18
**Inhibitors**	**Concentration (mM)**	**Relative Activity (%)**
Control	-	100
β-ME	5	55
DTT	5	60.7
EDTA	5	9.5
IAA	5	88
PMSF	5	49

The proteolytic activity was decreased by all the inhibitors (5mM) tested (Table 3). When exposed to EDTA, the enzyme activity was reduced by 90.5% indicating that the active site of the enzyme requires critical metal ions that were chelated by EDTA. This observation indicates that the enzyme is a metalloprotease that requires metal cofactors for activity. The protease activity was decreased by 45% in the presence of β-ME which suggested that the enzyme was denatured by the breakage of at least one disulfide bond necessary for maintaining the active form of the enzyme. DTT and PMSF both induced moderate inhibition, and IAA had a small inhibitory effect.

In the published literature, the synthesis of protease and corresponding enzyme activity have been reported in various strains of *Bacillus* bacteria under different pH and temperature conditions. Glucose is widely recognized as the preferred carbon source, while yeast extract is considered as the optimal nitrogen source for efficient enzyme production. Most of the isolated strains indicate that the production of enzymes is optimized at a temperature of 50 °C and an alkaline pH level. provides a compilation of several species that are known to produce protease, along with the corresponding optimal conditions for the synthesis of protease [25, 26].

The media’s composition, as well as the growth and fermentation factors given to the bacterial strain, have a significant impact on the expression of proteases. The *Bacillus subtilis* BSP was also greatly affected by the conditions of pH, temperature, metals, surfactants, and inhibitors that were tested. The results and responses to these factors are correlated with previous studies reviewed by Sharma et al. in 2017 [27]. In their study, they observed an increased level of protease production (2450 U/ml) in *Bacillus pseudofirmus* AL-89 when 60 g/l glucose was added. Additionally, an increase in glucose concentration resulted in a slight reduction in enzyme production, with the optimum level reported to be a 1% (w/v) concentration in most studies. Various carbon sources such as glucose, lactose, galactose, and starch have also been reported to result in maximum levels of protease production by *Bacillus aryabhattai K3* when used as carbon sources in the medium [27]. Different nitrogen sources, such as peptone, tryptone, potassium nitrate, and yeast extract phosphate at a concentration of 1%, have also been found to have an effect on protease production, as reported for the *Rheinheimera strain*. (NH4)2HPO4 has also been found to be the best nitrogen source for protease production by *A.oryzae* 637, and ammonium sulfate has been reported to be the best nitrogen source for protease production by *Bacillus sp.* strain AS-S20-I. The incubation time for protease production varies for each strain [27]. For example, protease production by *Bacillus pumilus* UN-31-C-42 starts 16 h after incubation, increases gradually, and reaches a maximum at about 28 h. For *B. subtilis* PE11 and *B. licheniformis* LBBL-11, maximum growth and enzyme production are observed after 2 days. *Bacillus sp.* APP1 produces the maximum titre of protease after an incubation period of 2 days. The maximum protease production from *B. subtilis*, and *B. licheniformis* is recorded after 72 h. Temperature is a critical parameter that needs to be controlled and varied for maximum cell growth and enzyme production, as it differs widely among different microorganisms [27]. The optimum temperature requirement for alkaline protease production also varies. For example, the optimum temperature for protease production by *B. licheniformis*, *B. coagulans*, and *B. cereus* is reported to be 30 °C, while a lower optimum temperature of 25 °C has been reported for *B. circulans*, *Microbacterium sp*., and 28 °C for *B. cinerea*. A temperature of 37 °C has been reported as the optimal temperature for protease production by a number of *Bacillus* species such as *B. amovivorus*, *B. proteolyticus* CFR3001, *B. aquimaris* VITP4, and *B. subtilis* strain Rand. On the other hand, a temperature of 40 °C has been reported to be the best for the production of protease by *Bacillus sp*. 2–5 and *B. licheniformis* GUS1. A high optimum temperature of 50 °C has been reported for *Bacillus sp*. strain APP1 and *B. subtilis* BS1. Various metal ions and reagents have been reported to influence the activity of proteases. For example, Ca + 2, Mg + 2, and Mn + 2 ions positively regulate the enzyme activity of alkaline protease from *B. circulans*, *Pseudomonas thermaerum* GW1, and *B. licheniformis* MP1. Isopropanol, methanol, and benzene increase the activity of protease from *Pseudomonas thermaerum* GW1 [27]. This indicates that, several factors, such as the pH of the production medium, ionic strength, temperature, and various surfactants, influence the performance of proteases.

## Conclusions

In this study, the thermophilic, protease-producing strain *Bacillus subtilis* BSP was isolated, and the production of the enzyme was optimized using both traditional (OVAT) and statistical response surface approaches. A 1.6-fold (295 U/mL) improvement in protease yield was attained using response surface methods in comparison to the OVAT analysis’s results (184 U/mL). In the form of a small number of distinct experimental setups, the Box-Behnken design produced useful information. It was also quite beneficial to see how various elements interact and have an impact on response surface plots. The *Bacillus subtilis* BSP strain protease has an ideal pH of 8.0 and a preferred temperature of 50 °C. Metal ions Ca^2+^ and Fe^2+^ were added, which enhanced the protease activity. It is the first time a *B. subtilis* protease has been reported to show stability against high concentrations of 10% SDS and 10% H_2_O_2_ making this enzyme possibly relevant for pharmaceutical and detergent industrial applications.

### Electronic supplementary material

Below is the link to the electronic supplementary material.

Supplementary Material 1

Supplementary Material 2

Supplementary Material 3

## Data Availability

All data is included in the manuscript, and necessary data is uploaded as supplementary files. The datasets generated for 16 S RNA of Bacillus subtilis BSP strain is available in the [GenBank (accession number EF644419.1 Bacillus subtilis strain BSP 16 S ribosomal RNA-like gene, partial sequence] at NCBI repository, online web https://www.ncbi.nlm.nih.gov/nucleotide/EF644419.1.

## Abbreviations

BSP: *Bacillus Subtilis* Protease
DMSO: Dimethyl Sulfoxide
DTT: Dithiothreitol
β-ME: β-mercaptoethanol
DTT: Dithiothreitol
EDTA: Ethylenediaminetetraacetate
IAA: Iodoacetamide
PMSF: Phenylmethylsulfonyl Fluoride
ANOVA: Analysis of Variance
SDS: Sodium Dodecyl Sulfate
RSM: Response Surface Methodology
