# Supplementary figures and images for: Characterization of a thermostable protease from Bacillus subtilis BSP strain
Source: BMC Biotechnol. 2024 Jul 15;24:49. doi: 10.1186/s12896-024-00870-5 (PMC11247832; doi:10.1186/s12896-024-00870-5)

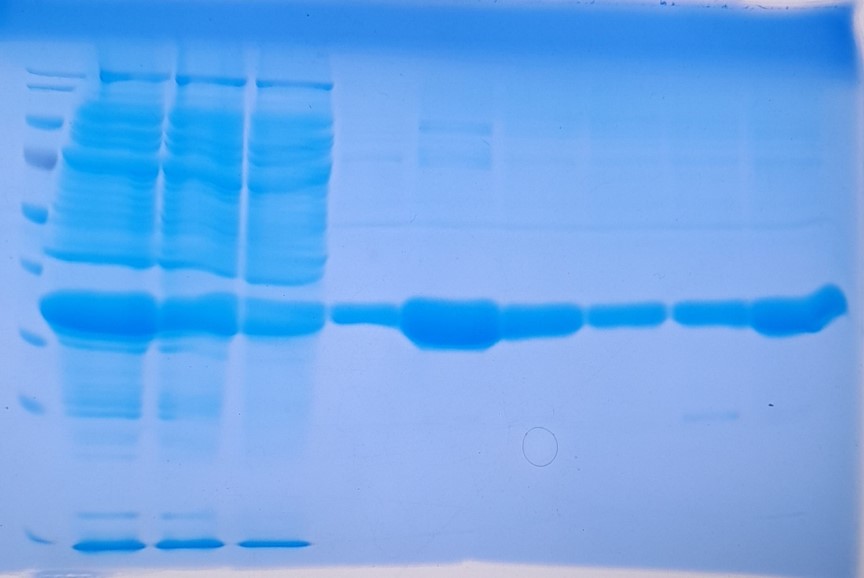

Supplement: Supplementary file 1 — Supplementary Material 1 [file 12896_2024_870_MOESM1_ESM.jpeg]

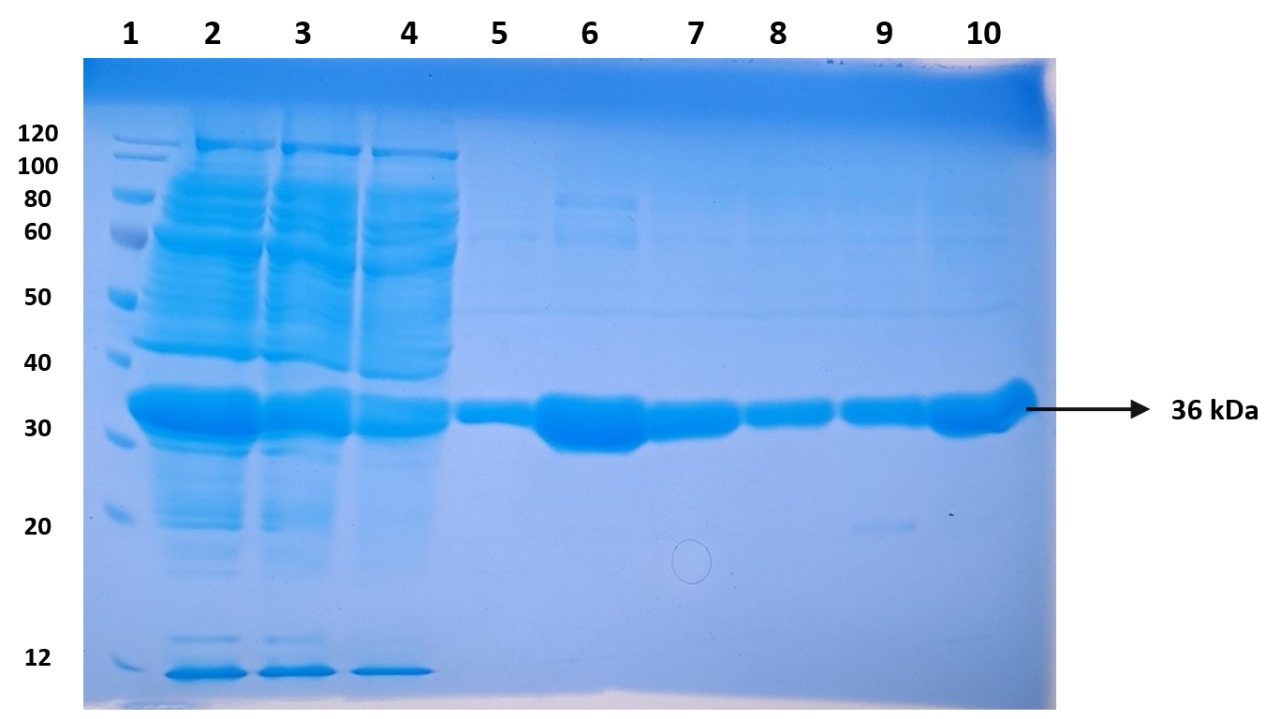

Supplement: Supplementary file 2 — Supplementary Material 2 [file 12896_2024_870_MOESM2_ESM.jpeg]
