# Supplementary material for: Characterization of a thermostable protease from Bacillus subtilis BSP strain
Source: BMC Biotechnol. 2024 Jul 15;24:49. doi: 10.1186/s12896-024-00870-5 (PMC11247832; doi:10.1186/s12896-024-00870-5)
Supplement: Supplementary file 3 — Supplementary Material 3 [file 12896_2024_870_MOESM3_ESM.docx]

**GenBank (accession number EF644419.1 of *Bacillus subtilis* BSP**

**>EF644419.1 Bacillus subtilis strain BSP 16S ribosomal RNA-like gene, partial sequence**

**AGAGTTTGATACCTGGCTCAGGACGAACGCTGGCGGCGTGCCTAATACATGCAAGTCGAGCGGACAGATG**

**GGAGCTTGCTCCCTGATGTTAGCGGCGGACGGGTGAGTAACACGTGGGTAACCTGCCTGTAAGACTGGGA**

**TAACTCCGGGAAACCGGGGCTAATACCGGATGCTTGTTTGAACCGCATGGTTCAAACATAAAAGGTGGCT**

**TCGGCTACCACTTACAGATGGACCCGCGGCGCATTAGCTAGTTGGTGAGGTAATGGCTCACCAAGGCGAC**

**GATGCGTAGCCGACCTGAGAGGGTGATCGGCCACACTGGGACTGAGACACGGCCCAGACTCCTACGGGAG**

**GCAGCAGTAGGGAATCTTCCGCAATGGACGAAAGTCTGACGGAGCCACGCCGCGTGAGTGATGAAGGTTT**

**TCGGATCGTAAAGCTCTGTTGTTAGGGAAGAAACAGTACCGTTCGAATAGGGCGGTACCTTGAACGGTAC**

**CTAACCAGAAAGCCACGGCTAACTACGTGCCAGCAGCCCGCGGTAATACGTAGGTGGCAGCGTTGTCCGG**

**AATATTTGGGCGTAAAGGGCTCGCAGGCGGTTTCTTAAGTTTGATGTGAAAGCCCCCGGCCTCAACCGGG**

**AAGGGTCTTGGGAAACTGGGGAACTGAGTGCAGAAGAAGAAAGTGGAATCACGNTGTTGCGGTGAATGCG**

**TNAAATGTGAGGACCCCAGTGCGAGGCCGGAATTAGATTCCCTGTGTTGTTCACGGCCGTAAACGGATTG**

**GTGGTTAGTGTTTGGGGGTTCCGCCCCTTAGTGCTGCAGCTACGCANTAAGACTCCGCCTGGGAGACGTT**

**CGCAGACTGAACTCAAGGGATTGACGGGTCCGCACAAGCGTTGAGCATGTGGTTAATTCGAAGCAACGCG**

**AAGAACCTTACCAGGTCTTGACATCTTCTGACAATCCTAGAGATAGAACGTCCCCTTCGGGGGCAGAGTG**

**ACAGGTGGTGCATGGTTGTCGTCAGCTCGTGTCGTGAGATGTTGGGTTAAGTCCCGCAACGAGCGCAACC**

**CTTGATCTTAGTTGCCAGCATTCAGTTGGGCACTCTAAGGTGACTGCCGGTGACAAACCCGGAGGGAAGG**

**GTGGGGAATGAACGTTCAAAATTCATTTCAATGCCCCCCCTTTAATGAACCTTGGGCTTAACAACAACGT**

**GCATACAAGGGGACGGGGGAGCGGGGAGGGCAGCGAAACCGCGAGGTTAAGCCAATCCCACAAATCTGTT**

**CTCAGTTCGGATCGCAGTCTGCAACTCGACTGCGTGAAGCTGGAATCGCTAGTAATCGCGGATCAGCATG**

**CCGCGGTGAATACGTTCCCGGGCCTTGTACACACCGCCCGTCACACCACGAGAGTTTGTAACACCCGAAG**

**TCGGTGAGGTAACCTTTTAGGAGCCAGCCGCCGAAGGTGGGACAGATGATTGGGGTGAAGTCGTAACAAG**

**GTAGCCGT**

**Amino acid Sequence of thermostable protease protein**

MNKRAMLGAIGLAFGLMAWPFGASAKEKSMVWNEQWKTPSFVSGSLLKGEDAPEELVYRYLDQEKNTFQLGGQARERLSLIGKQTDELGHTVMRFEQRYRGIPVYGAVLVAHVNDGELSSLSGTLIPNLDKRTLKTEAAISIQQAEMIAKQDVADAVTKERPAAEEGKPTRLVIYPDGETPRLAYEVNVRFLTPVPGNWIYMIDAADGKVLNKWNQMDEAKPGGGQPVAQTSTVGVGRGVLGDQKYINTTYSSYYGYYYLQDNTRGSGIFTYDGRNRTVLPGSLWADDNQFFASYDAAAVDAHYYAGVVYDYYKNVHGRLSYDGSNAAIRSTVHYGRGYNNAFWNGSQMVYGDGDGQTFLPFSSGIDVVGHELTHAVTDYTAGLVYQNESGAINEAMSDIFGTLVEFYANRNPWDWEIGEDIYTPGIAGDALRSMSDPAKYGDPDHYSKRYTGTQDNGGVHTNSGIINKAAYLLSQGGVHYGVSVTGIGRDKMGKIFYRALVYYLTPTSNFSQLRAACVQAAADLYGSTSQEVNSVKQAFNAVGVY


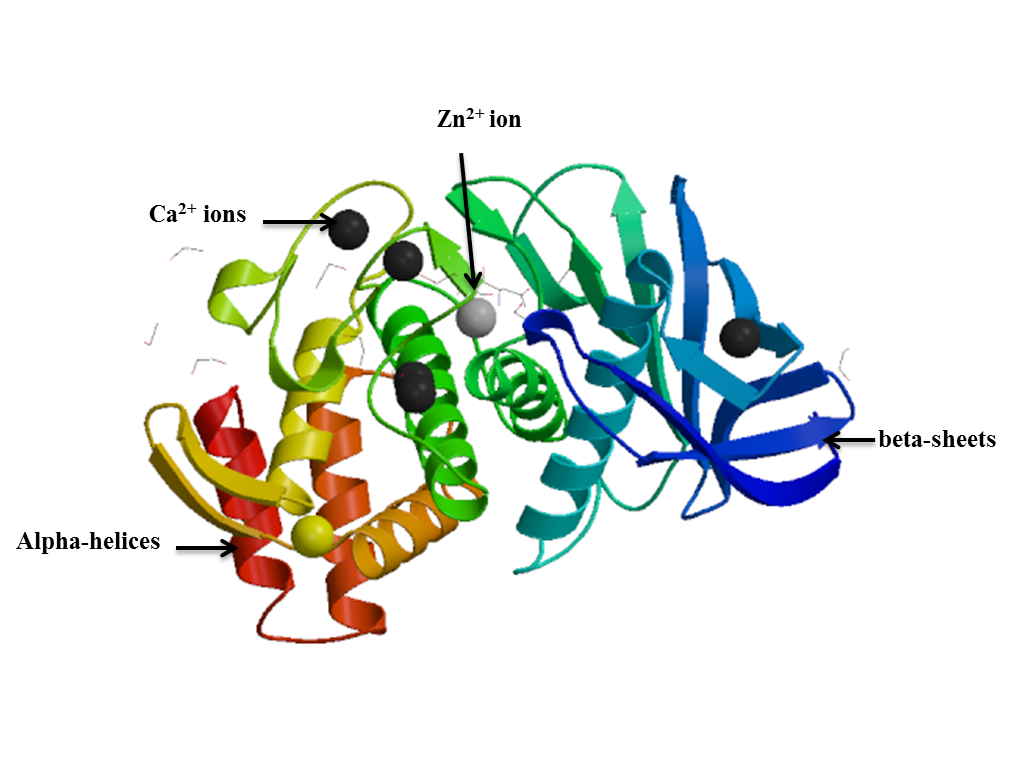


Supplementary Fig. The above figures represent predicted 3D structure of BSp-Mpr protease enzyme by Swiss model workspace structure prediction tool. [Secondary structure analysis of the metallprotease using SOPMA Expasy where (Hh) Alpha helix 35.16%; ( Ee) Extended strand 25.82%; ( Tt) Beta turn 11.36%; ( Cc) Random coil 27.66%.
